# Vaccination against COVID-19 — risks and benefits in children

**DOI:** 10.1007/s00431-023-05380-8

**Published:** 2024-01-02

**Authors:** Alasdair P. S. Munro, Christine E. Jones, Saul N. Faust

**Affiliations:** 1 NIHR Southampton Clinical Research Facility and Biomedical Research Centre, University Hospital Southampton NHS Foundation Trust, Southampton, UK; 2 Faculty of Medicine and Institute for Life Sciences, University of Southampton, Southampton, UK

**Keywords:** COVID-19, Vaccination, Immunisation, MIS-C, PIMS-TS, Long COVID

## Abstract

Countries in Europe and around the world have taken varying approaches to their policies on COVID-19 vaccination for children. The low risk of severe illness from COVID-19 means that even small risks from vaccination warrant careful consideration. Vaccination appears to result in a decreased risk of severe illness including the paediatric multi-system inflammatory syndrome known to be associated with COVID-19. These risks have already decreased significantly with the emergence of the Omicron variant and its subvariants, and due to widespread population immunity through previous infection. There is a relatively high risk of myocarditis following second doses of mRNA vaccines in adolescent males, although the general course of this condition appears mild.

*  Conclusion*: COVID-19 vaccination only provides a transient reduction in transmission. Currently, insufficient evidence exists to determine the impact of vaccination on post-acute COVID syndromes in children, which are uncommon.

**Table:** 

**What is Known:** *• Vaccines against COVID-19 have significantly reduced morbidity and mortality around the world.* *• Whilst countries have universally recommended vaccines for adults and continue to recommend them for vulnerable populations, there has been more variability in recommendations for children.*	
**What is New:** *• In the setting of near universal existing immunity from infection, the majority of the initial benefit in protecting against severe illness has been eroded.* *• The risks of myocarditis following mRNA vaccination for children is low, but an important consideration given the modest benefits.*

**What is Known:**

*• Vaccines against COVID-19 have significantly reduced morbidity and mortality around the world.*

*• Whilst countries have universally recommended vaccines for adults and continue to recommend them for vulnerable populations, there has been more variability in recommendations for children.*

**What is New:**

*• In the setting of near universal existing immunity from infection, the majority of the initial benefit in protecting against severe illness has been eroded.*

*• The risks of myocarditis following mRNA vaccination for children is low, but an important consideration given the modest benefits.*

## Introduction

The impact of the COVID-19 pandemic has been significantly mitigated by the development of highly effective vaccines which provide robust protection against severe disease and death [1]. Whilst the benefits of vaccination for high-risk groups such as older adults and the immunocompromised are uncontroversial, there has been active discussion around the benefits of vaccinating those who are at the lowest risk of severe illness, predominantly children. Initial emergency authorizations for new vaccines were only for those aged 16 years or older, and subsequent authorizations for younger age groups have come in a stepwise fashion; initially for those aged 12 years and older, then 5 years and older, and most recently for infants 6 months and older. The youngest infants (e.g., under 6 months) are at highest risk of hospitalisation with SARS-CoV-2 infection [2] and maternal vaccination in pregnancy can provide protection to those not yet eligible for vaccination themselves through transplacental transfer of maternal antibody [3]. This strategy is not discussed further in this review. Different countries have taken different approaches for approving or recommending vaccination for children, demonstrating the finer balance in the risk to benefit ratio of vaccinating children due to their extremely low risk of serious illness.

This article will review the various considerations in determining the potential benefits and costs of vaccinating children against SARS-CoV-2 and how these changed during the latter years of the pandemic. The focus is solely on vaccines available for use in children.

## Benefits

### Severe disease

Although initially offering protection against wild type virus, the most important function of vaccines for SARS-CoV-2 is to protect against severe COVID-19. Observational studies suggest that vaccination of children provides a substantial reduction in the risk of hospitalisation from COVID-19. A study of 255,936 children aged 5 to 11 years in Singapore during the omicron period estimated protection from 2 doses of BNT162b2 to be 82.7% (95% CI 74.8% to 88.2%) [4]. A retrospective study from Italy of 5 to 11-year-olds from January to April 2022 found protection against severe COVID-19 (mainly hospitalisation) to be 41.1% (95%CI 22.2% to 55.4%) [5]. A systematic review estimated protection from hospitalisation overall to be 75.3% (95%CI 48–90%), but was unable to estimate protection against death as the event rate was too low [6].

Several additional factors warrant consideration. Although the relative impact is large, because severe illness is so rare in children, the absolute benefit is very small. The MRC Biostatistics Unit COVID-19 working group estimated that as of December 2022 the infection hospitalisation rate for children aged < 5 years was 0.018% (1 in 5500) and for children aged 5 to 14 was 0.000087% (1 in 1 million) [7]. Most of the risk of severe illness falls on a small cohort of children with significant comorbidities, including life limiting conditions. Of the 15 children admitted in one London hospital for severe COVID-19 between December 2020 and January 2022, 11 had significant comorbidities including malignancy, primary immunodeficiency and prematurity [8]. A birth cohort study from Scotland found that 49% of all children and young people admitted with COVID-19 had at least one chronic condition recorded [9]. Of all COVID-19 deaths occurring in England between March 2020 and December 2021, 75.3% had an underlying condition such as severe neurodisability and severe immunocompromise such as cellular immunodeficiency [10]. Any attempt to determine the benefit of vaccination to children must define the impact on otherwise healthy children as well as those who are high risk of more severe disease, likely comprising only around 8% of the paediatric population [11]. This difference has been acknowledged in most countries where COVID-19 vaccine recommendations differ greatly depending on the presence or absence of risk factors in children. Of great importance for current consideration is that almost all children have also been exposed several times to SAS-CoV-2 and so have infection-derived immunity. The levels of protection estimated in these previous studies against a baseline of no population immunity will therefore be significantly reduced. It is estimated that over 98% of children aged > 1 year in England had been infected at least once as of December 2022 [7].

### Hyperinflammatory syndrome

An important potential benefit of vaccination for children against SARS-CoV-2 was protection against the multisystem inflammatory syndrome, known as PIMS-TS or MIS-C. For wild type virus in an unexposed population, this condition was estimated to occur following 1 in 3000–5000 SARS-CoV-2 infections in children [12] and was associated in some individuals with severe illness with myocardial dysfunction necessitating paediatric intensive care unit admission [13]. Studies suggest that vaccination reduces the risk of PIMS-TS following infection, with a Danish study estimating a relative risk of 0.11 (95% CI 0.01–0.83) [14]. However, there has been a substantial decline in the rates of PIMS-TS ever since the emergence of the Delta variant in 2021, and it has become extremely rare since the Omicron variant emerged [14, 15]. Whilst the reasons for this are not clearly defined, it may be because of new mutations in the spike protein of SARS-CoV-2 as well as population immunity gained through previous infection. As a result, the benefit of vaccination in reducing the risk of PIMS-TS is now reduced.

### Post-acute COVID-19 symptoms

A further possible vaccination benefit would be reducing the risk of post-acute COVID-19 symptoms, often referred to as long COVID. A large prospective cohort study in the UK aimed to describe the clinical phenotype and prevalence of long COVID symptoms in children and young people and a cohort study in Norway similarly investigated differences between SARS-CoV2 test positive and negative children and young people. Both studies found to have high rates of psychosocial factors in children with and without confirmed COVID-19 [16, 17]. In the UK study, only a very small number of children had symptoms longer than 3 months that impacted their daily lives [16]. Using the most generous definition of “any symptoms lasting beyond 12 weeks”, the ONS found rates to be low, with only 1% of 2- to 15-year-olds having any symptoms, and 0.6% with limiting daily activity. Rates were lower, but not statistically significant following a second infection at 0.6% and 0.4% respectively [18]. Currently, there is no evidence on whether vaccination impacts the risk of long COVID in children.

### Preventing transmission

The final potential benefit of vaccinating children for COVID-19 is in preventing transmission. When the vaccines were well matched to circulating virus prior to any vaccine approval in under 18s, this could have been an important factor in considering the risk benefit of vaccines in children. However, with viral evolution away from wild type, vaccines have become less effective at preventing transmission whilst still maintaining protection against the most severe illness. Vaccines can also prevent transmission by reducing the symptom and viral burden in an infected vaccinee, making them less infectious although there is no evidence thus far in children. Studies in adults show that vaccinated individuals are less infectious to other members of the household than unvaccinated, and unimmune individuals [19, 20]. We can extrapolate from these findings that children with immunity to SARS-CoV-2 are likely to be on average less infectious once infected than those who are naïve to infection or vaccine.

There is evidence from randomised trials that vaccination reduces the risk of symptomatic infection in children [21–24]. However, it is now well described in both adult and paediatric populations that the reduction in symptomatic infection wanes extremely rapidly alongside antibody levels, especially since the emergence of Omicron, and is minimal beyond 12 to 16 weeks [25, 26]. There is therefore only a short period of time within which it is possible to reduce transmission through vaccination. This makes the impact of primary immunisation of children small. Routine, periodic booster vaccination would be necessary to have a long-term impact on ongoing population transmission.

### Risks

#### Reactogenicity

The most common adverse event from vaccination is reactogenicity. Vaccines for COVID-19 are relatively reactogenic, however the reactogenicity profile is dose- and age-dependent. Children aged over 12 years tend to be administered adult doses of the vaccines and appear to experience the highest levels of reactogenicity compared to younger children [21–24, 27]. It is unclear whether the lower levels of side effects for approved COVID-19 mRNA vaccines in younger children are related to biological factors, or the lower dose administered. Side effects are typical of most other vaccines, with local effects of pain and swelling and systemic effects similar to a brief flu-like illness. Whilst this reactogenicity profile is considered tolerable, it is worth considering given the asymptomatic or mild nature of infection.

#### Myocarditis

The most common serious adverse event related to mRNA COVID-19 vaccines in young people is myocarditis. The prognosis of vaccine-related myocarditis appears to be favourable compared to viral myocarditis, with data from Hong Kong suggesting a 92% lower mortality risk [28]. The clinical course for most cases is mild, although abnormalities were still present in 54% of patients followed for at least 90 days since onset in a US study, with 68% cleared to return to physical activity [29]. Whilst many studies of the rates of myocarditis following COVID-19 vaccination provide population-wide estimates, the risks are highly dependent on age, gender, previous doses of vaccination, and possibly interval and dose of vaccine administered. Risks for females are only slightly elevated compared to expected rates and are higher in adolescent and young females than older females, with a risk around 2 to 3 per 100,000 doses [30–32]. The risk in males is higher, with a significantly increased risk between the ages of 12 and 30 which is highest after the second or third dose of vaccine. Rates also appear to be higher following mRNA-1273, although this has not been as widely used in children [30]. For adolescent males, rates after the second dose of BNT162b2 range from 6.7 to 15 per 100,000 [30–32]. There are limited data following third doses, but a Canadian study estimated a rate of 7 per 100,000 (observed to expected case ratio (OER) of 139.8 (95% CI 28.8–408.6) which was higher than following the second dose (OER 134.29, 95%CI 61.4 to 254) [31]. Data is more scarce for children aged under 12 years, but myocarditis appears to be less common in this age group, with one systematic review estimating an incidence of 1.8 per million [33]. It is unclear whether lower rates are a function of lower biological risk, a lower dose of vaccine, and/or reduced ascertainment in younger children. It is also unclear how previous antigen exposure through infection impacts risk, as most studies assume an infection naive cohort and do not address prior exposure from infection.

Many studies which attempt to compare the rates of myocarditis after vaccination versus COVID-19 infection overestimate the rates following infection, as the denominator is comprised only of cases detected via testing. It is well known that most infections of COVID-19 go undetected due to the low symptom burden, and so the true myocarditis rate following infection is likely to be several times smaller than estimated in these studies, by a factor of 5 to 10 [34].

#### Financial and opportunity costs

Other costs must also be taken into consideration. Given the risks to most otherwise healthy children from COVID-19 are low, a very large number of vaccinations are required to have a significant impact on a health resource utilisation. The costs of purchasing and administering the vaccines could quickly offset any economic gains from reduced health burden. In addition to financial costs are the opportunity costs of implementing additional vaccination programmes. At a time when many health systems are stretched, the staff and resources necessary to implement such programmes are considerable. This may also take staff away from implementing existing routine childhood immunisation schedules, which have already been detrimentally impacted by the pandemic [35].

A further consideration is the existing hesitancy from parents towards COVID-19 vaccination for children. Uptake of the primary schedule has been relatively low in many countries for a variety of reasons, including a perceived lack of necessity and many children having already been infected. Vaccine uptake for children aged 5 to 11 years in England only reached around 10% and is no longer available for this age group [36], in Germany 20% [37], and in Spain 32%, whilst children remain unvaccinated in Sweden where the vaccines are unavailable for this age group [38]. Despite being authorised in Germany for children aged 6 months to 5 years, COVID-19 vaccines have not been recommended for otherwise healthy children in this age group by the Standing Committee on Vaccination (STIKO) and also not by the Joint Committee on Vaccination and Immunisation (JCVI) in the UK. Hesitation towards COVID-19 vaccines could result in lower uptake of existing routine immunisation programmes (e.g., seasonal influenza) that were to be bundled together. Additional research is warranted to evaluate potential interactions with existing programmes. 

### Changing dynamics

At the time that vaccines first became available for COVID-19, there was little controversy that vaccination provided a significant benefit across the adult population, but even among adults there has been a shift in this dynamic over time. Many countries no longer recommend additional booster doses of vaccination for younger adults (e.g. aged less than 50, 60 or 65 years) without additional risk factors such as comorbidities or being at high risk for exposure [39]. This is due to the success of the initial vaccination campaign and the presence of a high degree of existing population from both immunisation and infection. In addition, the emergence of Omicron changed the dynamics in the setting of a less virulent but more transmissible variant of concern [40].

These dynamics have also changed for children. Early uncertainties around the increased risk of myocarditis following mRNA vaccines caused some hesitation in recommending vaccines for children as the risk appeared to increase with decreasing age (later it became clear that the risk was highest in adolescence up to age 30 years) [41]. In addition, at the time vaccines became available for the younger paediatric population, they remained largely immune naive. Now the population attack rate in children is estimated to be over 98%, with the majority of children having been infected more than once [7]. The greatest benefits from vaccination occurred prior to the first antigen exposure, and with each subsequent exposure the absolute benefits conferred from vaccination reduce. This has also had a significant impact on the benefits of protection against the multisystem inflammatory syndrome. Together, the current data would suggest that although the risks of vaccination are lower than had initially been feared, the benefits from vaccination in otherwise healthy children have also reduced over time from what was originally a relatively low baseline.

Whilst in the USA the CDC recommends COVID-19 vaccines for everyone over the age of 6 months, in the UK and European Union, COVID-19 vaccination is generally reserved for children aged 5 or over with significant co-morbidities who have an increased risk of severe COVID-19, and in some countries for children living with someone who has a weakened immune system. In practice, clinicians are advised to check national recommendations.

## Conclusion

The low risk of severe illness in otherwise healthy children means that even small risks of vaccination must be taken into consideration. Most of the potential benefit to be conferred from vaccination in preventing severe illness and or PIMS-TS/MIS-C has been minimised due to existing immunity from infection, and lower rates of hyper inflammatory response due to existing immunity and viral evolution. Any potential benefit in preventing viral transmission is marginal and short lived. In the setting of widespread existing population immunity through infection, the significant financial and opportunity costs of implementing further vaccination programmes may offset any benefit provided from transient increased immunity for otherwise healthy children. For children with significant comorbidities, there is a much larger absolute reduction in risk provided by periodic vaccination which is the basis of most current national public health recommendations.
